# Scientific Evidence in Public Health Decision-Making: A Systematic Literature Review of the Past 50 Years

**DOI:** 10.3390/ijerph22091343

**Published:** 2025-08-28

**Authors:** Emmanuel Kabengele Mpinga, Sara Chebbaa, Anne-Laure Pittet, Gabin Kayumbi

**Affiliations:** 1 Institute of Global Health, Faculty of Medicine, University of Geneva, 1202 Geneva, Switzerland; emmanuel.kabengele@unige.ch; 2 Faculty of Biology and Medicine, University of Lausanne, 1011 Lausanne, Switzerland; sara.chebbaa@unil.ch; 3 The Alan Turing Institute, London NW1 2DB, UK; gkayumbi@turing.ac.uk

**Keywords:** scientific evidence, public health, decision making, policy-makers, determinants, systematic review

## Abstract

Background: Scientific evidence plays a critical role in informing public health decision-making processes. However, the extent, nature, and effectiveness of its use remain uneven across contexts. Despite the increasing volume of literature on the subject, previous syntheses have often suffered from narrow thematic, temporal, or geographic scopes. Objectives: This study undertook a comprehensive systematic literature review spanning 50 years to (i) synthesise current knowledge on the use of scientific evidence in public health decisions, (ii) identify key determinants, barriers, and enablers, (iii) evaluate implementation patterns, and (iv) propose future directions for research and practice. Methods: We adopted the PRISMA model (Preferred Reporting Items for Systematic Reviews and Meta-Analyses). Moreover, we researched three large databases (Web of Science, Embase, and PubMed), and this study focused on articles published in the English and French languages between January 1974 and December 2024. Studies were analysed thematically and descriptively to identify trends, patterns, and knowledge gaps. Results: This review reveals a growing corpus of scholarship with a predominance of qualitative studies mainly published in public health journals. Evidence use is most frequently analysed at the national policy level. Analyses of the evolution of scientific production over time revealed significant shifts beginning as early as 2005. Critical impediments included limited access to reliable and timely data, a lack of institutional capacity, and insufficient training among policy-makers. In contrast, enablers encompass cross-sector collaboration, data transparency, and alignment between researchers and decision-makers. Conclusions: Addressing persistent gaps necessitates a more nuanced appreciation of interdisciplinary and contextual factors. Our findings call for proactive policies aimed at promoting the use of scientific evidence by improving the accessibility of health data (addressing the absence or lack of data, as well as its reliability, timeliness, and accessibility), and by training decision-makers in the use of scientific evidence for decision making. Furthermore, our findings advocate for better alignment between the agendas of healthcare professionals (e.g., data collection), researchers (e.g., the selection of research topics), and decision-makers (e.g., expectations and needs) in order to develop and implement public health policies that are grounded in and informed by scientific evidence.

## 1. Introduction

Perhaps more than any other discipline, the science of public health is a discipline of deliberations and, above all, of decision making [1,2,3]. Whether it concerns prevention programmes, hospital planning, training initiatives, epidemic and pandemic control measures, resource allocation, project and activity evaluation, or interventions during natural disasters and humanitarian crises, the practice of public health requires decision making processes.

The success and effectiveness of such decisions depend not only on the means deployed to achieve the objectives but also on the consideration of scientific evidence that may justify the choice of a particular decision, priority, or option over others [4,5].

Beyond the ineffective policies they may produce, ignorance, lack of awareness, and the non-use of evidence can lead to high rates of morbidity and mortality, restrictions in access to care, infringements of patients’ rights, significant economic and social costs associated with diseases, the non-participation of affected communities, and even the loss of credibility and trust in health systems [6,7,8,9].

It is undoubtedly due to this critical importance that the Evidence-Based Public Health movement emerged and developed, which Jenicek [10] defined as the conscientious, explicit, and judicious use of the best available evidence in decisions concerning the health of communities and populations, namely, in the areas of health protection, disease prevention, and the maintenance and improvement of health.

From its origins, in some place in the 1960s–70s, this movement has experienced various developments in response to the challenges faced by global public health, such as the emergence of new epidemics and the resurgence of old ones (HIV and tuberculosis), the introduction of new technologies in prevention and care (telemedicine, eHealth, artificial intelligence, etc.), the issue of social determinants and lack of access to care, climate change and the epidemiological transition, mental health crises, and health workforce shortages, as well as violence and armed conflicts.

Several examples help illustrate the importance of using scientific evidence in public health decision making. One of the most emblematic cases concerns tobacco control policies, particularly in the United States. This case has become widely recognised due to the *Reports of the Surgeon General*, which played a pivotal role in raising national awareness about the health risks of smoking. These reports helped shift the issue from being perceived primarily as a matter of individual or consumer choice to being understood as a matter of epidemiology, public health, and population-level risk, affecting both smokers and non-smokers alike [11].

Equally emblematic is the second case, which concerns the study of health inequalities among British civil servants. The findings from this research had a significant impact on public policy, influencing measures aimed at improving working conditions, promoting mental health in the workplace, and supporting broader social policies designed to reduce socio-economic disparities [12]. More recently, scientific evidence has been widely used in shaping responses to the COVID-19 pandemic [13].

Overall, these developments have led to substantial scientific production concerning the use of evidence in public health decision making, making the synthesis of these findings highly important in several respects. Indeed, our study synthesises fifty years of scholarship on the use of scientific evidence in public health decision making.

First, without such syntheses, decisions and interventions are likely to be inappropriate, ineffective, or even harmful to individuals and communities. Second, decision-making processes can be hampered by prolonged periods of deliberation. Finally, it is worth noting that policies underpinned by robustly synthesised evidence enhance public trust in health systems.

When viewed from a temporal perspective, the use of scientific evidence in public health decision making has already been the subject of several knowledge syntheses, most of which are limited. Their limitations stem either from narrow datasets, specific populations, short study periods, particular types of review, restricted thematic scopes, or limited geographic coverage.

For instance, the literature review conducted by Kneale et al. (2017) on the use of evidence in local public health decision making is narrow in scope, and its findings cannot be readily applied to other contexts [14]. Similarly, the review by Goyet et al. two years earlier focused solely on Cambodia [15].

The reviews conducted by the teams of Campbell et al. (2018) on increasing the use of research in public health policy and programmes, as well as by Innvaer et al. (2002) on policy-makers’ perceptions of their use of evidence, also illustrate the narrowness of databases and a focus on a specific subpopulation [16,17].

Regarding the thematic content of the reviews, limitations also exist. Norton et al. (2011) systematically examined research showing how public health decision-makers use evidence, drawing on materials from 15 qualitative studies and 3 surveys [18]. Meanwhile, Oliver et al. (2014) focused solely on barriers and enablers to evidence use by policy-makers [19].

The short time frames of prior reviews also restrict their conclusions. Kneale et al. (2017), for example, limited their scoping review to publications from 2010 only [14], and similarly, Masood et al. (2018) reviewed the use of research in public health policy over a brief period of 2010–2016 [20].

Additionally, the scopes of reviews vary considerably in terms of thematic focus. Some syntheses concentrate mostly on decision-makers’ perceptions of their use of evidence [16], while others look exclusively at barriers and obstacles to evidence use [17].

Finally, the knowledge syntheses produced also differ greatly according to their design. Alongside systematic reviews conducted according to rigorous guidelines [14,16], there are scoping reviews, narrative reviews, rapid reviews, and predictive studies [14,16,21,22], whose strength and generalisability require cautious interpretation.

It is precisely these limitations that motivated the present study, which differed from previous work in three key respects: a long historical outlook of 50 years; use of the broadest available databases (PubMed and, especially, Web of Science); and a robust, tested systematic review methodology with the following objectives:

(i) To synthesise knowledge on the use of scientific evidence in public health decisions;
(ii) To identify the determinants, barriers, and facilitators;
(iii) To contribute to the improvement of evidence-based public health decisions;
(iv) To evaluate the implementation of evidence;
(v) To define perspectives for future research on this topic.

These objectives stem from three research questions highlighted in this introduction and which can be formulated as follows:

- What is the current state of knowledge on scientific production concerning the use of evidence in public health decision making? Specifically, what are the main characteristics of this output in terms of volume, research themes, main geographic centres, leading contributors, publication channels, types of study design, etc., over the period under review?
- What are the obstacles, barriers, and/or facilitators to such use, or, more precisely, what are the organisational, structural, or contextual factors that influence, in one way or another, the use of evidence?
- Finally, what actions can be undertaken by actors and institutions to enhance the use of evidence in the formulation, implementation, and evaluation of public health policies, i.e., in public health practice?

Answers to these questions will be important for policy-makers, health professionals, research institutions, and other stakeholders, including international funding bodies engaged in global health. For the latter, the findings will help guide and direct their investments towards programmes and activities that are robustly evidence-informed.

Health professionals will gain insight into the determinants of successful or unsuccessful interactions with policy-makers, which are also central to the credibility and effectiveness of their interventions. With these findings, research institutions and investigators will also obtain guidance on knowledge gaps and future research directions in this domain. As for decision-makers, the recommendations arising from this study, as well as the future research prompted by it, will be of direct relevance to them.

Before addressing these questions, it is important to present the theoretical framework underpinning this study, as well as its overall structure.

## 2. Theoretical Framework and Study Structure

This study falls within the field of research on the use of scientific evidence in public health. It is grounded in a theoretical framework that encompasses the foundational paradigm of research in this domain; the nature of the problem concerning the gap between scientific evidence and its use in public policy; the explanatory theoretical models addressing this gap and their critiques; and the major challenges currently facing research on the use of scientific evidence in public policy.

The fundamental paradigm of research on the use of scientific evidence posits that public decisions, interventions, and actions must be grounded in scientific data to be legitimate [23,24]. 

Without going into detailed exposition, this paradigm has faced several critiques, including the difficulty of producing robust scientific evidence in certain contexts (e.g., pandemics, humanitarian crises, widespread violence, and complex emergencies); the failure to incorporate local and community knowledge; and the predominance of Western models and methodologies [25,26,27].

For some scholars, the core issue lies in the non-use of scientific evidence in public health decision making due to a variety of factors. This problem is characterised by the gap between the existence of evidence and its lack of use, underuse, or disregard. Understanding this gap entails identifying its explanatory causes as well as potential solutions [20,28]. 

With regard to the theoretical models, numerous explanatory theoretical models have been proposed over the years to account for the use or non-use of scientific evidence, each with its own limitations. One of the most cited is Weiss’s model of research utilisation, which argues that evidence use is more of a cumulative process of knowledge building rather than a direct or linear translation of evidence into action. However, this model has been criticised for not sufficiently accounting for contextual factors, timeframes (short- vs. medium-term use), and the complexity of decision-making processes, which are also shaped by political interests, institutional memory, and unique local contexts. In contrast, other scholars have focused on realist evaluation approaches, which aim to explain how and why evidence may or may not be used by policy-makers [29,30,31,32,33].

It should also be noted that the research on the use of scientific evidence in public policy continues to face several major challenges, such as

(i) Epistemological challenges, related to divergent understandings of foundational concepts such as “evidence” and what constitutes “research production” and “research use” [23,29,34].
(ii) Methodological challenges, linked to the complexity and heterogeneity of data, difficulties in contextualising or generalising results, and the misalignment between the production of evidence and political agendas [35].
(iii) Finally, the use of scientific evidence can face practical and ethical challenges. Among the practical barriers are financial constraints, a lack of or poor-quality data, fragmented data sources, and weak transmission mechanisms between evidence producers and decision-makers. Additionally, certain public health decisions based on evidence may raise risks of human rights violations if not handled ethically [36,37,38].

**Figure 1:**
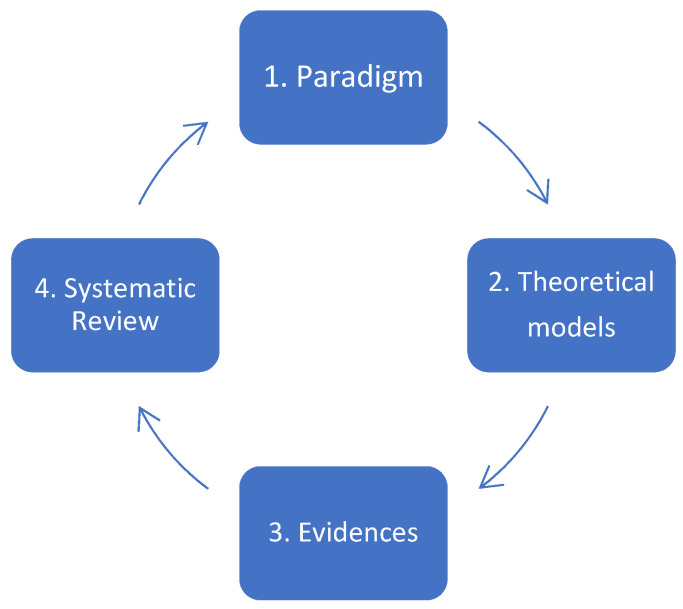
A schematic diagram of the theoretical framework.

Figure 1 shows a schematic diagram of the theoretical framework.

The application of this theoretical framework has generated a body of knowledge on the use of evidence in public health decision making, which we analysed through a descriptive systematic literature review.

## 3. Materials and Methods

This literature review adopted a systematic approach to establish the state of knowledge on the use of scientific evidence in public health decision making. The different stages of this study follow the guidelines of the Preferred Reporting Items for Systematic Reviews and Meta-Analyses (PRISMA 2020) [39].

### 3.1. Protocol

The protocol was drafted and validated with the support of the library team at the University of Geneva and subsequently registered on PROSPERO (CRD420251025428).

### 3.2. Search Strategy

The research question was translated into a search strategy using keywords. The search strategies were pre-tested, adapted to each database, and then validated with the assistance of the University of Geneva library team. The literature search was carried out in November 2024. It was applied to three databases: PubMed, Web of Science, and Embase. The keywords were designed to capture the concept of evidence use (“Use of evidence” OR "Utilization of evidence” OR “Application of evidence” OR “Evidence-based public policy” OR “Evidence-based policy” OR “Evidence-based public health policy” OR “Evidence-based public policies” OR “Evidence-based policies” OR “Evidence-based public health policies” OR “Evidence-informed policies” OR “Evidence-informed policy” OR “Use of research evidence” OR “Utilization of research evidence”), the concept of policy making (“Policy making” OR “Politics” OR “Policy” OR “policymaker*” OR “policy-maker*” OR “policymaking” OR “policy-making” OR “policy” OR “policies” OR “public health decision*”), as well as the concept of public health (“Public Health” OR “Public Health Administration” OR “Health Policy” OR “Public health” OR “national health” OR “governmental health” OR “health field*” OR “health policy” OR “health policies”), and, finally, the concept of positive, negative, or neutral determinants (“determinant*” OR “factor*” OR “barrier*” OR “facilitator*” OR “limitation*” OR “strengthen*” OR “support*” OR “hinder*” OR “facilitate*” OR “promote*”) (File S1: Details of Boolean search string for each database).

#### 3.2.1. Data Extraction

We identified eligible articles by using the PRISMA 2020 flow diagram. The first two authors independently scanned all titles and abstracts selected in their search, and articles that were assessed as not relevant were discarded. So were duplicates.

#### 3.2.2. Exclusion Criteria

We then applied the following exclusion criteria: articles published before 1974, articles without an abstract, articles written in a language other than French or English, press releases, editorials, technical reports, symposium/conference abstracts or articles, magazine or newspaper articles, glossaries, commentaries, books and book chapters, project descriptions, descriptive reports, technical notes, viewpoints, position papers, tutorials, and methodology articles.

#### 3.2.3. Data Analysis

We developed a data extraction categorisation to apply to the final inventory of articles (File S2). First, we extracted the article’s year of publication, the first author, and their institutional affiliation. The latter was determined based on information from the database (PubMed), article content, and the corresponding author’s email address. Affiliations were categorised as follows: “university”, “government”, “foundation”/“think tank” or “private company”, “international organisation”, or “other”. This enabled the identification of the first author’s institutional affiliation country.

Two authors (E.K. and S.C.) independently assigned the categories of journals, topics, domains, and study designs before cross-checking the data. Rare disagreements on categorisation were resolved through discussion until consensus was achieved.

Regarding journal types, they were categorised as “biomedical”, “care-focused”, “public health”, “natural sciences”, “human and social sciences”, or “mixed”.

Themes were assigned to articles according to their title and abstract. The available themes were ”evidence production”, ”use”, ”implementation”, ”evaluation”, “translation”, “collaboration”, “resource allocation”, and “capacity building”.

In our study, the term

- “Evidence” does not reside only in the world where science is produced; it emerges in the political world of policy making, where it is interpreted, made sense of, and used, perhaps persuasively, in policy arguments [40].
- “Evidence production” means scientific evidence production.
- “Use” refers again to the utilisation of scientific evidence.
- “Implementation” is the carrying out of planned, intentional activities that aim to turn evidence and ideas into policies and practices that work for people in the real world [41,42].
- “Evaluation” is the systematic process to determine merit, worth, value, or significance [43].
- “Translation” is associated with knowledge utilisation and refers to the use of knowledge in practice and decision making by the public, patients, health care professionals, managers, and policy-makers [44].
- “Collaboration” is a core activity of a collaboration to share resources and capabilities that make the participators work closely together to create mutually beneficial outcomes [45].
- “Resource allocation” is an aggregation of the functions required to track and manage all resources related to production. These resources include labour, machines, tools, fixtures, materials, and other entities, such as documents that must be available in order for work to start at the operation [46].
- “Capacity building” is the development of knowledge, skills, commitment, structures, systems, and leadership to enable effective health policies to build capacity for public health [47].

To determine the domains of the studies, several combined criteria were used, especially elements from the title, abstract, and journal of publication. Two broad domains emerged: on the one hand, “public health” (“preventive”) and, on the other hand, “medicine” (“curative”).

As for the study design, the article categorisation was based on information derived from the title, abstract, and journal of publication. These categories were: “qualitative study”, “case study”, “focus group”, “Delphi”, “descriptive quantitative”, “cohort”, “case–control”, “randomised controlled trial”, “socio-political analysis”, “ethical/moral”, “legal/judicial”/“medico-legal”, “historical”, “philosophical”, “economic”, “theological”, “narrative review”, “rapid review”, “scoping review”, “realist review”, “systematic review”, “meta-analysis”, or “mixed-methods study”.

Next, four variables enabled us to establish the geographical context of the studies. These included the number of study sites (“single-site”, “multi-site”, or “global”), the scope of the study (“local”/“community”, “provincial”, “national”, “international”, or “regional”), the continent where the study took place, and the level of development of the study country (“developed” or “developing”).

Finally, we extracted from the abstracts the determinants presented by the authors as barriers and/or facilitators to the use of evidence, when they were explicitly described as such. The frequency of occurrence of these determinants across the analysed articles was weighted by the number of times each determinant was mentioned so as to highlight the relative importance of each one.

## 4. Results

**Figure 2:**
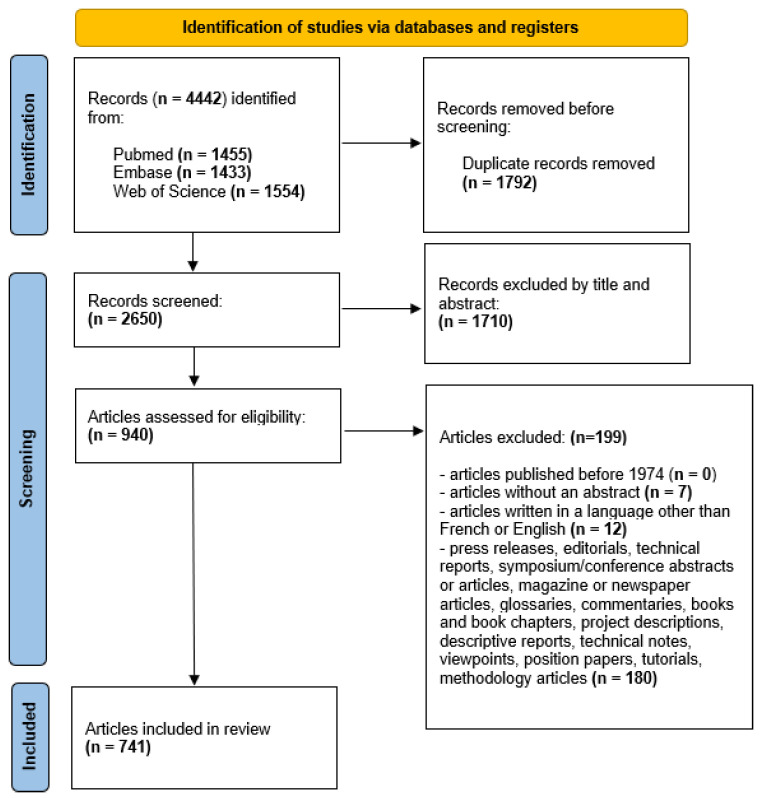
PRISMA 2020 flow chart diagram of study selection process.

**Figure 3:**
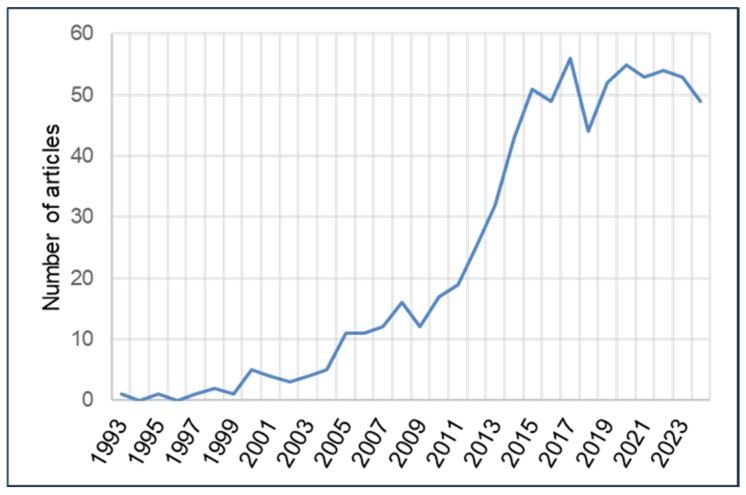
Evolution of annual scientific production from 1974 to 2024.

**Figure 4:**
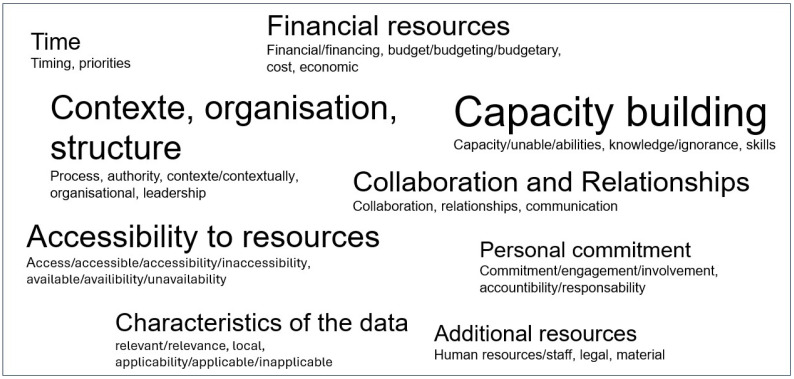
Determinants, facilitators, and barriers to the use of evidence. In this word cloud, the occurrence of determinants across the analysed articles was weighted by the number of times they were mentioned.

We present the results of our study in Figure 2, Figure 3 and Figure 4 and Table 1, Table 2, Table 3, Table 4, Table 5 and Table 6.

### 4.1. Application of PRISMA Model (PRISMA 2020)

This search yielded 4442 articles, among which 2208 duplicates were removed. A further 1710 articles were excluded as they did not match the thematic scope of our research question. We then applied the exclusion criteria to the remaining 940 articles. Subsequently, we applied the data extraction categorisation to the final 741 articles (File S2). Figure 2 depicts a flow diagram of the study selection process, as per the PRISMA guidelines (PRISMA 2020) (see also the PRISMA checklist (File S3)).

### 4.2. Characteristics of Scientific Production

#### 4.2.1. Annual Volume of Scientific Production

The evolution of the annual scientific production is shown in Figure 3. The 50-year window between 1974 and 2024 includes the publication of 741 articles, which were sorted according to the year of publication. In the data extracted from the 741 articles included in this study, we observed an exponential increase in published articles, moving from 1 in 1993 to 49 articles in 2024, with a peak observed in 2017.

#### 4.2.2. Types of Journals and Study Designs, Study Settings and Scopes, and Author Affiliations

The majority of these articles were published in English (99.5%). Most were published in public health journals (63.8%), followed by biomedical sciences (17.8%) and humanities and social sciences (10.5%) journals (Table 1).

In terms of study designs (Table 1), the literature was predominantly composed of qualitative studies (32.4%), followed by case studies (18.9%), quantitative studies (11.2%), and sociopolitical analyses (8.4%). The authors were mainly affiliated with universities (78.1%), compared with governments (10.1%), private foundations and organisations (6.1%), and NGOs (3.5%).

Apart from studies labelled as “international” (27.9%), the continents that contributed most of the articles analysed were Europe (18.2%), North America (17.3%), Africa (14.3%), Oceania (14.3%), and Asia (9.2%) (Table 2). The lead authors were most frequently based in the United Kingdom (19.2%), the United States (19%), Australia (14.3%), Canada (11.9%), and Nigeria (3.8%) (Table 3).

**Table 1:** Types of journals and study designs.

	Frequency	Percentage
Type of publication journal		
Public health	473	63.8%
Biomedical	132	17.8%
Humanities and social sciences	78	10.5%
Mixed	54	7.3%
Natural sciences	3	0.4%
Care-focused journals	1	0.1%
Study design		
Qualitative studies	240	32.4%
Case studies	140	18.9%
Focus groups	11	1.5%
Delphi Studies	4	0.5%
Descriptive quantitative studies	83	11.2%
Cohort studies	1	0.1%
Case–control studies	0	0.0%
Randomised controlled trials	7	0.9%
Socio-political analyses	62	8.4%
Ethical/moral analyses	1	0.1%
Legal, medico-legal, and juridical analyses	3	0.4%
Historical analyses	4	0.5%
Philosophical analyses	2	0.3%
Economic analyses	1	0.1%
Psychological analyses	1	0.1%
Narrative reviews	61	8.2%
Rapid reviews	5	0.7%
Scoping reviews	19	2.6%
Realist reviews	3	0.4%
Systematic reviews	38	5.1%
Meta-analyses	1	0.1%
Mixed-methods studies	54	7.3%

**Table 2:** Study settings and scopes.

	Frequency	Percentage
Continent of the study		
South America	18	2.4%
Asia	68	9.2%
Oceania	79	10.7%
Africa	106	14.3%
North America	128	17.3%
Europe	135	18.2%
International	207	27.9%
Study scope		
Local/community	11	1.5%
Regional (EU, AU, WHO, Africa, etc.)	49	6.6%
Provincial	62	8.4%
International	218	29.4%
National	401	54.1%

**Table 3:** Authors’ affiliations.

	Frequency	Percentage
Institutional affiliation of the first author		
University	579	78.1%
Government	75	10.1%
Foundation, think tank, or private organisations	45	6.1%
International organisation	26	3.5%
Other	16	2.2%
Country of the first author’s institutional affiliation (top ten)		
United Kingdom	142	19.2%
USA	141	19.0%
Australia	106	14.3%
Canada	88	11.9%
Nigeria	28	3.8%
Switzerland	24	3.2%
Iran	21	2.8%
South Africa	12	1.6%
Lebanon	11	1.5%
The Netherlands	10	1.3%

#### 4.2.3. Main Contributors

Table 4 presents the main authors who contributed to the publications.

**Table 4:** Main authors.

Main Authors	Frequency
Uneke, C. J. et al.	14
El-Jardali, F. et al.	8
Nabyonga Orem, J. et al.	6
Lavis, J. N. et al.	5
Smith, K. E. et al.	5
Zardo, P. et al.	5
Armstrong, R. et al.	4
Khalid, A. F. et al.	4
Oliver, K. et al.	4
Onwujekwe, O. et al.	4
Waqa, G. et al.	4

### 4.3. Content

#### 4.3.1. Domains and Themes of Study

The themes addressed by these studies (Table 5), in order of magnitude, pertained to the use of evidence (42.5%), implementation (33.9%), evidence production (25.9%), and translation (15.5%) (Table 5).

**Table 5:** Domains and themes of study.

	Frequency	Percentage
Theme		
Production	192	25.9%
Use	315	42.5%
Implementation	251	33.9%
Evaluation	65	8.8%
Translation	115	15.5%
Collaboration	13	1.8%
Resource allocation	37	5.0%
Capacity building	32	4.3%
Domain		
Medicine	35	4.7%
Public health	706	95.3%

#### 4.3.2. Determinants (Barriers and/or Facilitators)

Finally, the context, the internal organisation and structure of health services, institutions, and/or health programs; the competencies of human resources; access to the necessary resources, including financial resources; and personal commitment and appropriate timing between the availability of evidence and the problems to be addressed, constituted the fundamental determinants of the use—or non-use—of evidence in public health (Figure 4).

### 4.4. Analysis of Trends in Scientific Production over Time

Over the study period, scientific production has undergone a number of changes. First, although authors affiliated with universities consistently dominated throughout the entire period under review, we observed that starting from 2010 (the inflexion point), the proportion of publications authored by individuals affiliated with governmental institutions and those working in foundations, think tanks, or private companies increased by 871% for the former and 550% for the latter.

Similarly, in terms of study scope, a turning point appeared around 2010, marked by a shift from a national and international focus to a growing number of studies centred at the provincial and regional levels, with their proportions rising, respectively, by 277% (provincial) and 4700% (regional).

Along the same lines, while single-site studies continued to dominate the landscape of scientific production throughout the study period, the number of multi-site and global studies began to increase significantly after 2010.

Regarding publication channels, a shift can be identified slightly earlier, around 2005. Despite the continued predominance of public health journals as the primary outlets, we observed a notable increase in the number of articles published in biomedical journals starting in 2005.

Finally, when analysing thematic domains, no major changes were observed in the volume of publications across the two main areas. In other words, articles related to public health remained largely dominant throughout the entire study period. The ratio of the differences between the two types and their sum remained, respectively, at 75% between 1993 and 2009, and 92% between 2010 and 2024.

These trends are summarised in Table 6.

**Table 6:** The trends in scientific production between 1993 and 2024. The first column indicates relevant variables, while the second and third report the numbers of articles over the two intervals marked by the inflexion point around 2010.

Variables	1993–2009	2010–2024
Author affiliation		
Universities	72	507
Government institutions	7	68
Foundations, think tanks, and private companies	6	39
Study scope		
Provincial	13	49
Regional	1	48
Study setting		
Single site	54	397
Multi-site	16	151
Global	19	104
Type of journal		
Biomedical	29	48
Public health	102	425
Study domain		
Medicine	11	24
Public health	78	628

## 5. Discussion

In this study, we set out to investigate the use of scientific evidence in public health decision making. The findings of this review identified that the issue of evidence-based decision making in public health continues to attract growing interest. To the best of our knowledge, the findings revealed an absence of scientific production during the first 20 years of our study, across the languages and databases examined. This is not a unique characteristic of our study, as other authors have reported similar observations [48,49]. This phenomenon could be explained by the relatively recent emergence of interest in the subject.

Furthermore, while the results indicated a temporary negative impact of the COVID-19 pandemic on the volume of scientific production on this topic, the overall trend in production over time follows a steady increase. The COVID-19 pandemic has had various effects on the use of scientific evidence in public health decision making [50].

On the one hand, the pandemic revealed a politicisation and selective use of scientific evidence, which led to intense polarisation and tensions between knowledge producers and decision-makers. The rise and activities of groups labelled as conspiracy theorists illustrate this extreme politicisation [51].

Secondly, there was a noticeable erosion of public trust in institutions and policy directives. This loss of trust was accompanied by widespread misinformation, including the rapid dissemination of unverified data, notably in the form of fake news on social media platforms [52].

The speed of scientific publishing during this same period is another notable feature that may have contributed to the rapid uptake of evidence by decision-makers. As demonstrated in the study by Schonhaut, “There were 31,319 research articles on COVID-19 and 4287 on human influenza published during 2020. The median time to acceptance for COVID-19 was significantly shorter than that for human influenza (8 vs. 92 days). The median time to publication for COVID-19 articles was also shorter (12 vs. 16 days). Notably, 47.0% of COVID-19 research articles were accepted within the first week of submission, and 19.5% within one day” [53].

Beyond this dynamic, it is important to recall that key decisions in the management of the pandemic—such as vaccination strategies, mask mandates, the use of hand sanitiser, and lockdowns—were based on Western epidemiological models. These measures were often replicated and implemented as-is in contexts with markedly different demographic, epidemiological, political, and economic realities, without sufficient understanding, analysis, or testing by local decision-makers [54].

Finally, it is worth noting the increased visibility of public health professionals and researchers in the media and within pandemic response structures, which undoubtedly influenced decision-making processes during the course of the pandemic [55].

By and large, the observed increase in production trend is not specific to public health or to the issue of scientific evidence use in this field. Other literature syntheses in engineering research, public health, biomedical sciences, and accounting demonstrate the same pattern [49,56,57].

Several factors may explain this trend: the emergence and proliferation of new scientific publication channels, changes in publication processes, the “commercialisation” of academic publishing, technological advancements, and the expansion of research institutions (universities). It is reasonable to anticipate that the integration of artificial intelligence tools in scientific production will further reinforce this trend [58].

Regarding journal types and research domains, the majority of publications are found in public health journals, with a smaller proportion appearing in biomedical journals. These publications predominantly focus on preventive health (public health) and only marginally on curative health (medical). This distribution can be explained by the fact that biomedical journals primarily concentrate on clinical issues, experimental studies, and laboratory research, with limited attention paid to public health concerns. In contrast, public health decisions pertain to implementation science, health systems research, and policy evaluation.

Our findings indicate a predominance of qualitative study designs. This poses challenges for policy-makers, who tend to favour quantitative studies, a preference that is not exclusive to health sector decision-makers [59,60]. The prevalence of qualitative studies over empirical research complicates both the comprehension and utilisation of evidence by policy-makers, who are often required to communicate their decisions using numerical data. This reliance on quantitative metrics may hinder the effective use of scientific evidence in public policy [61,62]. Moreover, it should be recalled that for some, systematic reviews have become central in certain domains as a mechanism for translating evidence into policy, particularly in sectors beyond public health, such as public administration and transport policy [63,64].

The prevailing international/global scope of studies highlights that many qualitative investigations approach the subject from a global perspective. Given that health policies are often defined at the national level, the predominance of this research scope, as demonstrated in our study, is understandable. The underlying centralised administrative model raises concerns about the consideration of regional and community-specific particularities. It is regrettable that few studies have focused on the use of scientific evidence in decision making at the community level.

Our literature review indicates the existence of global disparities in knowledge production and its relevance to policy.

From a geographical perspective, the analysis of the geographical distribution of scientific production within our corpus supports the reliability of our methodology. The primary countries of affiliation for first authors include England, the United States, Australia, and Canada. While Anglo-Saxon countries dominate scientific research, questions arise regarding the applicability of such evidence to diverse cultural, social, and political contexts, particularly in the implementation of local policies in non-Anglo-Saxon political systems. A notable finding was the prominent contribution of Nigerian authors to research on this topic. This result stems from the work of Chigozie J Uneke’s team at the African Institute for Health Policy and Health Systems, facilitated by substantial investment in the establishment and operation of their research infrastructure [65].

The issue of inequalities in knowledge production is reflected in the dominance of research, funding, and publication structures by high-income, so-called “developed” countries, in contrast with those in the Global South. Salager Meyer points out that the existing disparity is also highlighted by the fact that 90% of important scientific research is published in 10% of journals, and while developing countries comprise 80% of the world’s population, only 2% of indexed scientific publications come from these parts of the world [66].

A deeper analysis of this asymmetry also reveals significant epistemological gaps. The predominance of Western paradigms and methodologies tends to marginalise local knowledge systems. Indigenous, experiential, or non-Western epistemologies are often dismissed as “anecdotal” or “unscientific”. Akena (2012) and Chanza (2013) examined the production of Western knowledge and its validation, imposition, and effects on indigenous people and their knowledge. The author argues that there is a relationship between knowledge producers and their motives and the society in which they live. This relationship influences what is considered “legitimate knowledge” in society, politics, and the economy in non-Western contexts [67,68].

Similarly, the legacy of Eurocentrism continues to affect knowledge production in the social sciences. Evidence produced in and about the Global North is assumed to be more “universal”, whereas evidence from or produced in the Global South is considered valid only for specific contexts (i.e., “localised”) [69].

Another major disparity relates to the influence of Western donors in setting priorities, allocating resources, and shaping the strategic directions of global health. The study by Nugent et al. highlights this imbalance by examining donor spending on noncommunicable diseases (NCDs) in developing countries between 2001 and 2008. The analysis reveals that less than 3 per cent of total development assistance for health (DAH)—that is, USD 503 million out of USD 22 billion—was allocated to NCDs in 2007–2008. Donor assistance for health targeting NCDs reached USD 686 million in 2008.

When compared in terms of disease burden, donors provided approximately USD 0.78 per disability-adjusted life year (DALY) attributable to NCDs in developing countries in 2007, in contrast with USD 29.90 per DALY for HIV/AIDS, tuberculosis, and malaria. The authors estimate that if donors were to provide even half the level of support per DALY for NCDs that they currently allocate to the three major infectious diseases, this would amount to nearly USD 4 billion in DAH for NCDs [70,71].

The other major disparity previously mentioned concerns cultural bias. For example, it is well established that over 85% of the global population resides in low- and middle-income countries where English proficiency is less common than in high-income countries. Such disparities may exacerbate the discrepancies in both producing and accessing scientific literature. This issue is particularly pertinent in critical care. For example, among the top four countries with the highest numbers of intensive care unit beds worldwide (the United States, Brazil, China, and Germany), only one has English as its native language [72].

Structural problems affecting scientific research in countries of the Global South must also be taken into account in the analysis of these disparities, as noted by Ciocca and Delgado, the major factors contributing to low scientific productivity are the limited access to grant opportunities, inadequate budgets, substandard levels of laboratory infrastructure and equipment, the high cost and limited supply of reagents, and the inadequate salaries and personal insecurity of scientists. The political and economic instability in several Latin American countries results in a lack of long-term goals that are essential to the development of science. In Latin America, science is not an engine of the economy. Most equipment and supplies are imported, and national industries are not given the incentives to produce these goods at home [73].

Overall, the production and use of scientific evidence at the global level remain shaped by scientific colonialism and neo-colonialism, the dominance and constraints imposed by funding structures, imbalances in data hierarchies, ineffective knowledge transfer, and the limited applicability of so-called universal solutions to health challenges in non-Western contexts.

Regarding the institutional affiliations of first authors, our results indicate that the majority of studies originate from universities. Indeed, universities remain the primary hubs for knowledge production worldwide. This could serve as a lever for evidence utilisation by policy-makers, as decision-makers place significant importance on the source of data as opposed to its quality [74,75,76].

The analysis of research themes reveals that few studies have focused on evaluation, collaboration, or competencies. Most research has concentrated on evidence utilisation and the application of knowledge and practices. This trend may be attributed to the lack of an evaluation culture [77,78]. It is important to note that, given the frequencies observed, a single article could address multiple themes.

This study highlights that only approximately one-third of the reviewed works provide information on the determinants (factors and/or barriers) influencing the use of scientific evidence in public health decision making. The results align with those of a systematic review on barriers to evidence utilisation in public policy more broadly [17,19,79]. A more detailed analysis of how evidence is marginalised, ignored, or used reveals that

(i) Evidence is often instrumentalised or disregarded depending on the political or institutional context. Centralised political systems, for example, are less conducive to research uptake, as power concentration limits pluralistic debate and reduces demand for evidence. In contrast, decentralised or federal systems foster greater use of research to legitimise and defend policy decisions [37,80].
(ii) Evidence may be used in ways that are more strategic than scientific, with policy-makers tending to rely more heavily on technical reports from international agencies than on scientific data generated at the community or local level [80].
  Indeed, the authors of those studies identify the most frequently cited obstacles as limited access to research, a lack of relevant studies, timing constraints/lack of opportunities for result application, and insufficient research literacy among policy-makers and other users.
(iii) Certain forms of scientific evidence are either adopted or dismissed depending on the influence of lobbyists within national decision-making bodies, particularly in policy areas such as drug regulation, tobacco control, and the food and pharmaceutical industries. International organisations can exert direct power through conditionality attached to aid or loans, or indirect power by setting norms and standards that national governments adopt [81,82].
(iv) The ability—or inability—to adapt and interpret knowledge in relation to local contexts can either facilitate or hinder the use of evidence from a technical standpoint [83].
(v) Finally, it is important to recall that the prevailing culture of evidence hierarchies—particularly the privileging of quantitative over qualitative data—can lead to the marginalisation of qualitative evidence in certain public health decisions. According to some authors, specific collaborative environments between researchers and policy-makers can help facilitate the use of evidence in decision-making processes [5].

With regard to the evolution of scientific production over time, a deeper understanding requires placing these developments within the historical trajectory of the evidence-informed decision-making movement. This history has evolved across several key phases. Prior to 1950, the dominant paradigm was that of rational planning in public health decision making. Between 1950 and 1970, there was increased reliance on the technological model and the use of scientific research, notably influenced by Carol Weiss’s model of research utilisation. The third phase, spanning from 1980 to 1990, was marked by the institutionalisation of evidence-based medicine (EBM), particularly through the foundational work of the Cochrane Collaboration. The period from 2000 to 2010 witnessed the rise of realistic evaluation and a growing emphasis on stakeholder engagement in decision-making processes. From 2010 to the present, new tools and approaches have emerged, including Big Data, artificial intelligence (AI), and a heightened focus on critical reflection and epistemological vigilance in public health decision making. The year 2010 represents a turning point, reflecting a broader shift towards inclusive decision-making frameworks. This period is characterised by greater involvement of foundations and think tanks and a stronger emphasis on integrating the data, needs, and perspectives of diverse stakeholders involved in shaping public health policies. These changes also reflect a growing epistemological awareness regarding the nature, quality, and relevance of evidence in contemporary public health governance [19,84,85].

It should be noted that these developments did not occur in a linear or sequential manner, but rather as part of a continuum marked by the accumulation and layering of knowledge.

### Strengths and Limitations

Beyond its internal coherence and external validity, our study presents several strengths. It is based on a broader corpus of materials than previous research on the subject.

Specifically, over 4000 references were initially collected, with approximately 800 articles analysed. The data collection was conducted using databases that are not only extensive but also multidisciplinary. Web of Science, for instance, encompasses more than 20,000 scientific journals and over one billion cited references across the domains of science, social sciences, arts, and humanities. Additionally, the long observation period (50 years, from 1974 to 2024) provides this review with a strong historical perspective for analysis.

However, this study also has certain limitations. A clear cultural bias exists, as the analysed works have been predominantly shaped by Anglo-Saxon culture, its healthcare system model, and its social and governance structures. The generalisability of the findings is, therefore, constrained when applied to other contexts, such as the majority of African healthcare systems (additional explanatory factors include low investment, etc.) [19,86,87,88].

In light of the specific historical, political, cultural, and economic contexts of Africa—as well as certain countries in Asia and Latin America—evidence-informed decision making must be understood and operationalised differently. This would require the following:

(i) Conceptual clarification and contextual adaptation of nosologies, that is, the definitions and classifications of diseases that are culturally and epidemiologically relevant to local health realities.
(ii) Methodologically, while randomised controlled trials (RCTs) are often considered the gold standard, incorporating experiential knowledge, including community narratives, traditional knowledge systems, and local health practices, may usefully complement formal scientific evidence.
(iii) In contexts where health information systems are weak or non-operational, efforts should be made to leverage routine data generated by community-based structures, and not to rely solely on academic or institutional data sources.
(iv) In cases where conflicts arise between the research agendas of donors, national or local governments, and community needs, the establishment of mediation and negotiation structures could help broker consensus and lead to politically and socially acceptable decisions, even in the absence—or in the presence of limitations—of conventional scientific evidence.

Finally, it would be beneficial to examine the funding sources and mechanisms behind the research leading to the analysed publications. Understanding these financial influences would help identify the interests of the funding bodies supporting these studies.

## 6. Future Research

This research allows for the identification of several avenues for future studies. The actions required to address the need to incorporate scientific evidence into public health decisions call for the design of national, and even regional, stakeholder partnership strategies founded upon (i) the establishment of frameworks, structures, infrastructures or research institutions, and mechanisms for political decision making based on evidence; (ii) the development of standards, law, or regulations governing scientific research and their harmonisation; and (iii) basic and continuing training to prepare researchers and policy-makers to work collaboratively. Regarding funders, research-funding institutions should reconsider their approaches to priority setting by aligning funding decisions with the pressing needs identified by national and local governments, as well as by the communities directly concerned.

Future research agendas might focus on the following objectives: (i) analysing the political, social, and cultural influences that explain the use or non-use of evidence; (ii) investigating the determinants that shape the thematic choices of international research-funding bodies and their impact on research priorities in different countries; (iii) improving the quality, accessibility, and timeliness of health data, especially in underserved areas, as well as ensuring interoperability across health information systems; (iv) identifying and testing effective strategies for translating evidence into concrete policies, overcoming implementation barriers, and assessing the impact of these strategies on health outcomes; (v) evaluating the role of universities in generating evidence and, especially, in facilitating its use in public policy; (vi) exploring the training needs of policy-makers to enable them to make better use of scientific evidence; and finally, (vii) developing robust data collection methods, fostering local research initiatives, and creating databases that support informed policy making; (viii) examining the alignment between research topics and findings, on the one hand, and policy-makers’ expectations and requirements, on the other; and (ix) bridging the existing gap between centres of knowledge (universities) and centres of decision making.

## 7. Conclusions

Our research highlights the existence of a science concerning the use of scientific evidence in public health decision making, for which the volume of scientific production is continuously increasing.

Interest in this topic originates predominantly from the Anglophone academic world and is reflected mostly in the production of qualitative studies, published largely in public health journals. Although public health decisions can be taken at various levels, most studies focus on the use of evidence at the national level. The synthesis we conducted on the themes covered by these studies indicates that this interest is primarily directed towards the use, implementation, and generation of evidence for public policy.

The analysis of trends in scientific production over time revealed significant shifts beginning in 2010, highlighting the importance of considering the historical contexts in which public health challenges emerge, as well as the necessity of evidence-based decision making to effectively address them.

Our findings underscore existing gaps that need to be addressed in future research, particularly the lack of coverage of evidence use at local, community, and regional levels. It is also crucial to keep in mind that the inherently interdisciplinary nature of the issue of evidence use in public health necessitates a deeper understanding of policy-makers’ needs with regard to (i) health data (absence or lack of data, data reliability, timeliness, and accessibility) and (ii) training on the use of scientific evidence for decision making. Aligning the agendas of health professionals (i.e., data collection), researchers (i.e., research priorities), and decision-makers (i.e., expectations and requirements) would enable the development of informed public health policies that meet the expectations and needs of both nations and communities. These avenues for research are essential to the formulation of recommendations, especially for certain continents facing fragile health systems, such as those in Africa.

The use of scientific evidence in public health decision making must be understood within the historical trajectory of the evidence-based movement (EBM) and the diverse contexts in which it is applied; our systematic review highlights its dynamic and evolving nature and underscores the pressing need for continued research in this field.

## Data Availability

This review utilised published studies that are available in the public domain.

## Supplementary Materials

The following supporting information can be downloaded at https://www.mdpi.com/article/10.3390/ijerph22091343/s1. File S1: Details of Boolean search string for each database; File S2: Main version of table used to extract data from included studies; File S3: The PRISMA checklist.
