# Supplementary material for: Scientific Evidence in Public Health Decision-Making: A Systematic Literature Review of the Past 50 Years
Source: Int J Environ Res Public Health. 2025 Aug 28;22(9):1343. doi: 10.3390/ijerph22091343 (PMC12469439; doi:10.3390/ijerph22091343)
Supplement: Supplementary file 1 [file ijerph-22-01343-s001.zip › ijerph-3769588-supplementary S1.pdf]

File S1- Details of Boolean search string for each database.

Database	Search strings	Results (n)(26.11. 2024)
PubMed	("Use of evidence"[tiab] OR "Utilization of evidence"[tiab] OR "Application of evidence"[tiab] OR "Evidence-based public policy"[tiab] OR "Evidence-based policy"[tiab] **OR "Evidence-based public health policy"[tiab]** OR "Evidence-based public policies"[tiab] OR "Evidence-based policies"[tiab] **OR "Evidence-based public health policies"[tiab] OR "Evidence-informed policies"[tiab]** OR "Evidence-informed policy"[tiab] OR "Use of research evidence"[tiab] OR "Utilization of research evidence"[tiab]**)** AND ("Policy Making"[Mesh] OR "Politics"[Mesh] OR "Policy"[Mesh:NoExp] OR policymaker*[tiab] OR policymaking[tiab] OR policy[tiab] OR policies[tiab] OR "public health **decision*"[**tiab]) AND ("Public Health"[Mesh] OR "Public Health Administration"[Mesh] OR "Health Policy"[Mesh] OR "Public health"[tiab] OR "national health"[tiab] OR "governmental health"[tiab] OR "health field*"[tiab] OR "health policy"[tiab] OR "health policies"[tiab]) AND (determinant*[tiab] OR factor*[tiab] OR barrier*[tiab] OR facilitator*[tiab] OR limitation*[tiab] OR strengthen*[tiab] OR support*[tiab] OR hinder*[tiab] OR facilitate*[tiab] OR promote*[tiab])	1'455
Embase	((((use OR utili?ation OR application) NEAR/2 evidence):ti,ab,kw) OR 'evidence-based public policy':ti,ab,kw OR 'evidence-based policy':ti,ab,kw OR 'evidence-based public health policy':ti,ab,kw OR 'evidence-based public policies':ti,ab,kw OR 'evidence-based policies':ti,ab,kw OR 'evidence-based public health policies':ti,ab,kw OR 'evidence-informed policies':ti,ab,kw OR 'evidence-informed policy':ti,ab,kw) AND ('decision making'/de OR 'politics'/exp OR 'policy'/de OR 'policymaker*':ti,ab,kw OR 'policymaking':ti,ab,kw OR 'policy':ti,ab,kw OR 'policies':ti,ab,kw OR 'public health decision*':ti,ab,kw) AND ('public health'/exp OR 'public health service'/exp OR 'health care policy'/exp OR 'public health':ti,ab,kw OR 'national health':ti,ab,kw OR 'governmental health':ti,ab,kw OR 'health field*':ti,ab,kw OR 'health policy':ti,ab,kw OR 'health policies':ti,ab,kw) AND ('determinant*':ti,ab,kw OR 'factor*':ti,ab,kw OR 'barrier*':ti,ab,kw OR 'facilitator*':ti,ab,kw OR 'limitation*':ti,ab,kw OR 'strengthen*':ti,ab,kw OR 'support*':ti,ab,kw OR 'hinder*':ti,ab,kw OR 'facilitate*':ti,ab,kw OR 'promote*':ti,ab,kw)	1433
WebOfScience	(((use OR utili?ation OR application) NEAR/2 evidence) OR "evidence-based public policy" OR "evidence-based policy" OR "evidence-based public health policy" OR "evidence-based public policies" OR "evidence-based policies" OR "evidence-based public health policies" OR "evidence-informed policies" OR "evidence-informed policy") AND ("policymaker*" OR "policymaking" OR "policy" OR "policies" OR "public health decision*") AND ("public health service" OR "public health" OR "national health" OR "governmental health" OR "health field*" OR "health policy" OR "health policies") AND ("determinant*" OR "factor*" OR "barrier*" OR "facilitator*" OR "limitation*" OR "strengthen*" OR "support*" OR "hinder*" OR "facilitate*" OR "promote*")	1554
